# Unmasking Wilson’s Disease Through Severe Psychiatric Manifestations: A Case Report

**DOI:** 10.7759/cureus.103993

**Published:** 2026-02-20

**Authors:** Filipe Dias, Ana Santos e Silva, Margarida Santos, Luísa Alvarenga, Beatriz Domingos, Sofia Sobral, Hipólito Nzwalo

**Affiliations:** 1 Internal Medicine, Unidade Local de Saúde do Litoral Alentejano, Santiago do Cacém, PRT; 2 Stroke Unit, Algarve University Hospital Center, Faro, PRT; 3 Faculty of Medicine and Biomedical Sciences, University of Algarve, Faro, PRT

**Keywords:** agitation management, copper metabolism, emergency department, kayser-fleischer rings, psychiatric symptoms, wilson disease

## Abstract

Wilson’s disease is an inherited disorder of copper homeostasis with highly variable clinical expression. In some patients, psychiatric syndromes dominate the early disease course and divert diagnostic reasoning toward primary mental illness, delaying etiologic treatment.

A 38-year-old woman was brought to the emergency department by police with onset of behavior change characterized by public disinhibition and intense motor agitation. Initial complete neurological assessment was limited by the presence of severe dysarthria. After initial treatment, the neurological evaluation disclosed the presence of extrapyramidal clinical manifestations. The presence of rings around the cornea was also documented on the ophthalmological examination. Laboratory studies revealed thrombocytopenia, mild aminotransferase elevation, profoundly reduced ceruloplasmin, and low serum copper. Brain magnetic resonance imaging demonstrated bilateral basal ganglia and thalamic hyperintensity with extension into the brainstem. Ophthalmologic slit-lamp examination confirmed the presence of Kayser-Fleischer rings, and molecular analysis identified a pathogenic ATP7B variant. Treatment with penicillamine combined with psychiatric stabilization was followed by gradual clinical improvement over subsequent weeks.

Acute behavioral and psychotic syndromes may represent the leading manifestation of Wilson’s disease. In young adults presenting with atypical psychiatric disturbances accompanied by dysarthria, extrapyramidal signs, and ophthalmologic abnormalities. High suspicion led to the diagnosis of Wilson’s disease, with early treatment leading to progressive improvement.

## Introduction

Wilson’s disease arises from genetically determined failure of hepatocellular copper transport, most frequently related to pathogenic variants in ATP7B, resulting in impaired biliary excretion and pathological copper accumulation in multiple organs [1,2]. Although traditionally conceptualized through its hepatic manifestations, the disease frequently extends beyond the liver, with neurological and psychiatric involvement that may dominate the clinical picture [1-3].

Psychiatric features are intrinsic to the disease spectrum rather than secondary phenomena. Both historical and contemporary series document manifestations ranging from affective disturbance and personality change to psychosis, behavioral dysregulation, and suicidal ideation [4,5]. When presentation occurs in emergency or psychiatric settings, organic etiologies may receive insufficient consideration, and Wilson’s disease may be misclassified as primary psychiatric illness, leading to clinically relevant diagnostic delay [2,5].

Neuroimaging contributes to the diagnosis, but it does not rule it out. While basal ganglia abnormalities are frequently observed, lesions may involve other structures, and the absence of well-known teaching signs does not preclude the diagnosis [6-9]. Large imaging cohorts indicate that delayed diagnosis correlates with larger central nervous system involvement, including brainstem and cortical regions, highlighting the importance of early diagnosis [6]. We report a case in which psychiatric disturbance was the dominant presenting feature and highlight the convergence of clinical examination, biochemical testing, ophthalmologic assessment, and neuroimaging that enabled the establishment of the diagnosis and initiation of treatment.

## Case presentation

A 38-year-old woman was brought to the emergency department by police after being found naked in a public setting. At presentation, she was unable to provide a coherent medical history. The patient exhibited intense psychomotor agitation with aggressive behavior, requiring physical restraint to ensure patient and staff safety. Communication was markedly limited due to severe dysarthria. Vital signs were within normal values, including blood pressure and heart rate. Cardiopulmonary examination was unremarkable. A brief neurological assessment, performed under suboptimal conditions, revealed equal and reactive pupils without nystagmus. Grayish rings around the corneas were visible on gross inspection. Deep tendon reflexes were symmetric, and plantar responses were equivocal.

Given the severity of agitation, pharmacological behavioral containment with antipsychotic medication was initiated.

Due to the patient’s inability to provide reliable information, a brief history was obtained from a neighbor. According to this source, the patient had been functionally independent until several months prior to admission, when progressive behavioral and functional decline became apparent. She reportedly lost her job at a food-processing facility due to difficulties performing manual tasks. Approximately two weeks before presentation, she developed escalating agitation, disorganized speech, and expressed suicidal ideation. The episode culminated in public behavioral disinhibition, prompting police involvement and transport to the emergency department.

Laboratory evaluation demonstrated thrombocytopenia, mild elevation of aspartate aminotransferase, markedly reduced ceruloplasmin levels, and low serum copper concentration. Serum ammonia and coagulation parameters were within normal limits. Cerebrospinal fluid analysis was unremarkable, including a negative multiplex PCR panel for neurotropic viruses. Abdominal ultrasound showed changes compatible with chronic liver disease.

**Figure 1:**
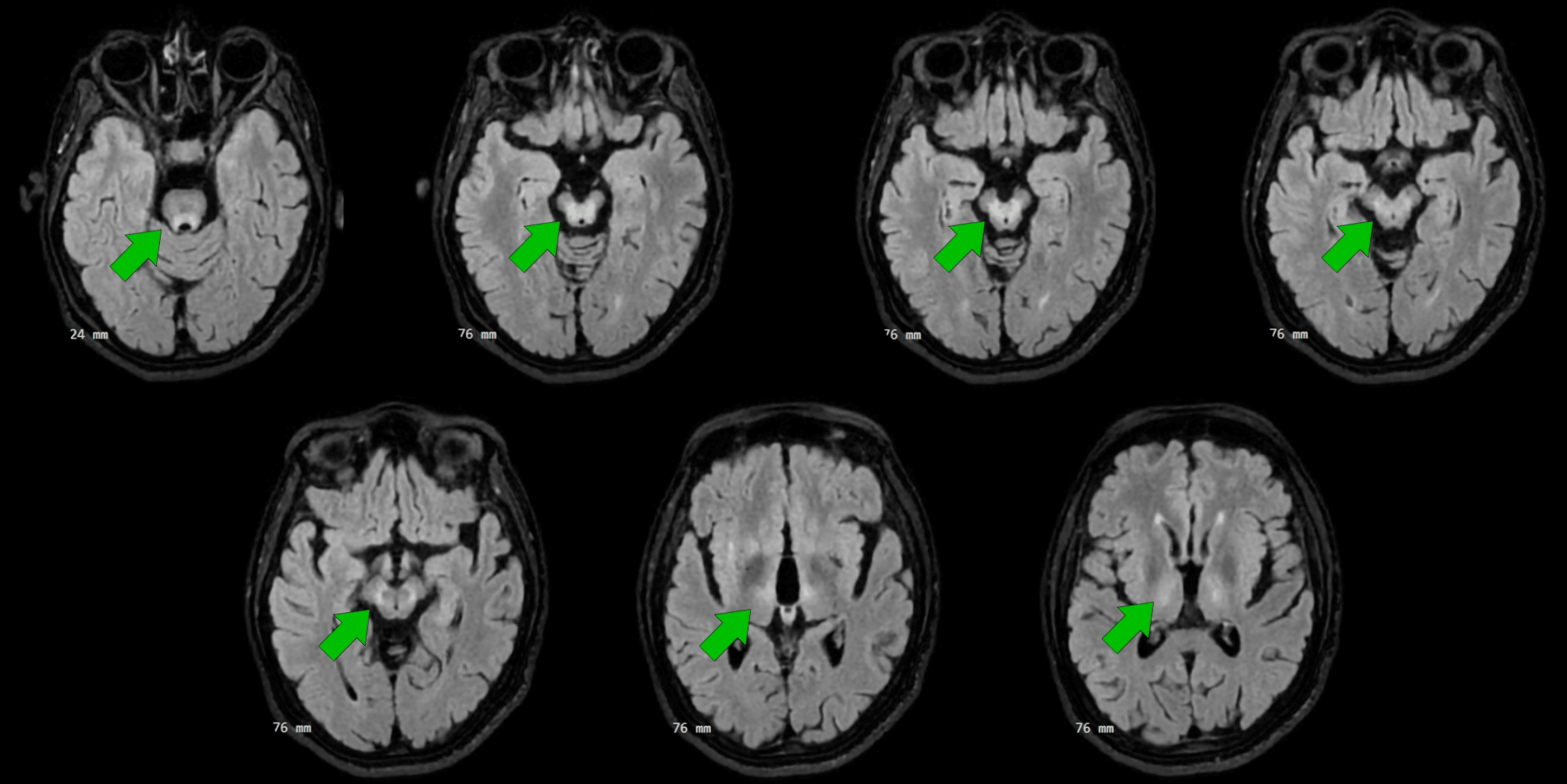
Fluid-attenuated inversion recovery (FLAIR) sequence cuts of brain magnetic resonance imaging showing bilateral symmetric hyperintensity of the anterior and middle thalami, extending through the cerebral peduncles into the pons and floor of the fourth ventricle

**Figure 2:**
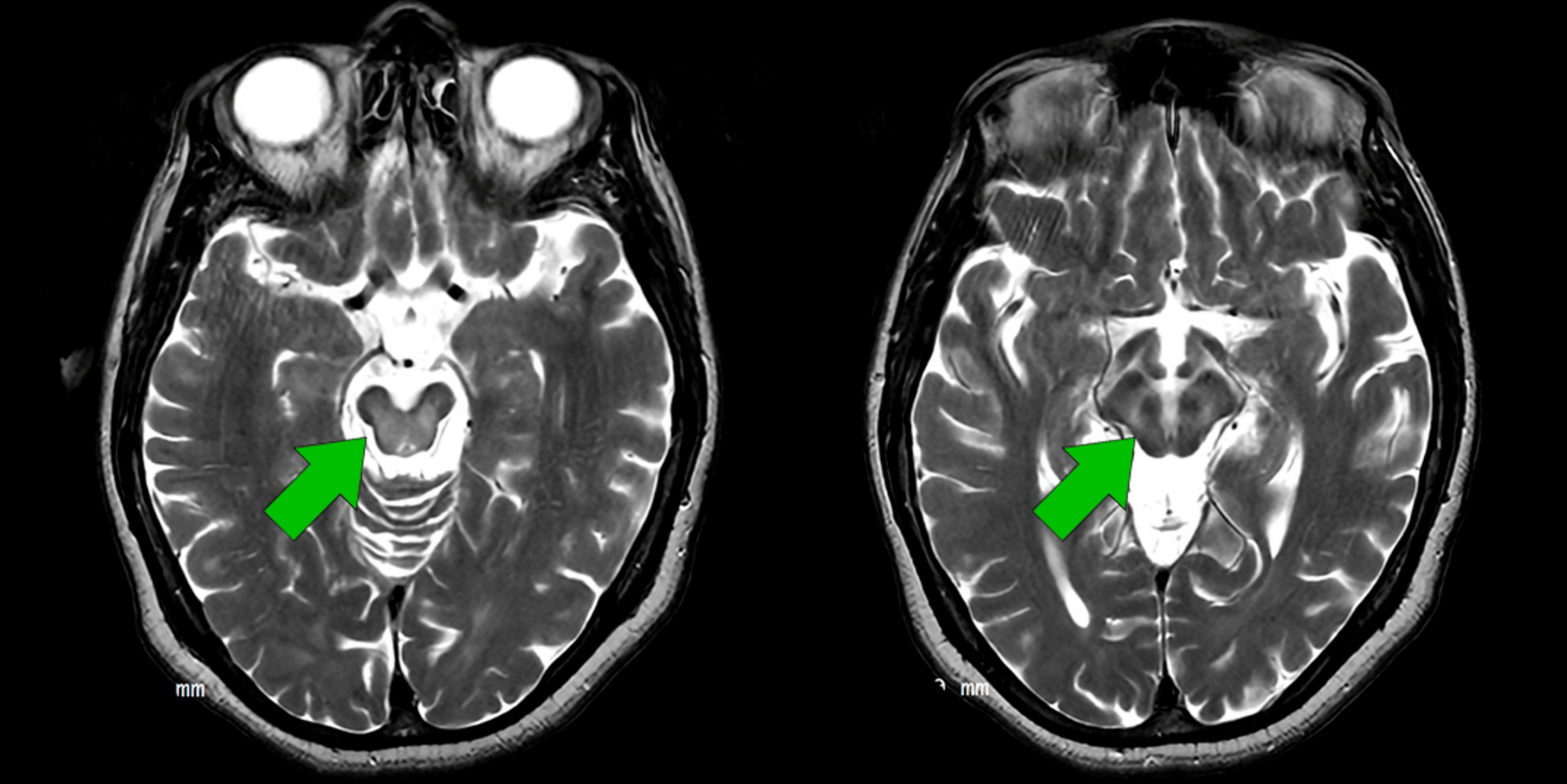
T2 sequence cuts of brain magnetic resonance imaging, with Giant Panda sign on the right

**Figure 3:**
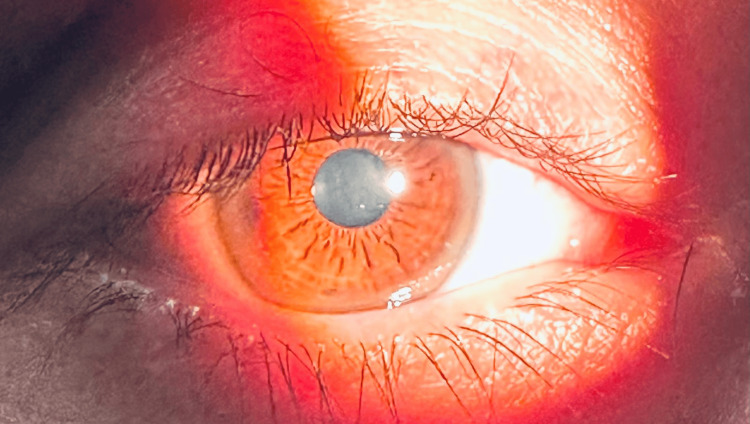
Slit-lamp photograph demonstrating Kayser–Fleischer rings

Brain magnetic resonance imaging revealed bilateral, symmetric hyperintensity on T2/fluid-attenuated inversion recovery (FLAIR) sequences involving the anterior and middle thalami, extending through the cerebral peduncles into the pons, with additional basal ganglia involvement (Figures 1, 2). Ophthalmologic slit-lamp examination confirmed the presence of Kayser-Fleischer rings (Figure 3). A summary of the most relevant laboratory and ancillary test results is presented in Table 1.

**Table 1:** Summary of relevant laboratory and ancillary test results FLAIR: fluid-attenuated inversion recovery, AST: aspartate aminotransferase, ALT: alanine aminotransferase, INR: international normalised ratio, APPT: activated partial thromboplastin time

Analysis	Result	Reference values
Complete blood count		
Platelet count	93,000 /µL	150,000–400,000 /µL
Liver and metabolic profile		
AST	60 U/L	0–35 U/L
ALT	15 U/L	0–35 U/L
Serum albumin	3.2 g/dL	3.5–5.2 g/dL
Serum ammonia	22 µmol/L	11–51 µmol/L
Coagulation studies		
INR	1.02	0.8–1.2
APTT	27.6 s	25–35 s
Copper metabolism		
Ceruloplasmin	<3 mg/dL	16–45 mg/dL
Serum copper	3.0 µmol/L	12.6–26.6 µmol/L
Cerebrospinal fluid analysis		
Appearance	Clear	Clear
Leukocytes	3 cells/mm³	<5 cells/mm³
Differential	No predominance	—
Albuminocytologic dissociation	Absent	Absent
Multiplex PCR panel (neurotropic viruses)	Negative	Negative
Neuroimaging		
Brain MRI – T2/FLAIR	Bilateral symmetric hyperintensity of the anterior and middle thalami, extending through the cerebral peduncles into the pons and floor of the fourth ventricle; discrete hyperintensity of the putamina	—
Brain MRI – T1	Corresponding hypointensity in affected regions	—
MR angiography	Normal intracranial arterial flow	—
Other imaging		
Cranial CT	No acute intracranial abnormalities	—
Abdominal ultrasound	Features compatible with chronic liver disease	—

Treatment with penicillamine was initiated at a total daily dose of 1,200 mg administered in four divided doses, in combination with antipsychotic medication for psychiatric stabilization.

Genetic analysis later identified a heterozygous pathogenic frameshift variant in ATP7B, together with two heterozygous variants of uncertain clinical significance.

Following partial behavioral stabilization with antipsychotic therapy, a more comprehensive neurological examination was possible. This revealed bradyphrenia, intention and postural tremor, choreiform movements, focal lingual dystonia, dysarthria, increased muscle tone predominantly affecting the lower limbs, and an unstable, broad-based gait without clear truncal or appendicular ataxia.

Over the subsequent weeks, the patient showed steady clinical improvement. At approximately three weeks after treatment initiation, neurological reassessment revealed residual but attenuated bradyphrenia, intention and postural tremor, focal lingual dystonia, mild dysarthria, lower limb rigidity, and an unstable but independent gait.

## Discussion

This presentation exemplifies a recurring diagnostic challenge: Wilson’s disease may manifest as a psychiatric emergency, and in such contexts the underlying metabolic disorder can be overlooked unless neurological features are actively identified and interpreted [4,5]. Contemporary guidelines emphasize that neuropsychiatric phenotypes are integral to the disease spectrum and that heterogeneity in presentation contributes substantially to delays in diagnosis and treatment [1-3]. In emergency settings characterized by limited history and immediate safety concerns, reliance on objective neurological signs becomes particularly important.

Psychiatric manifestations in Wilson’s disease encompass a wide range of syndromes. High-intensity agitation, behavioral disorganization, and psychotic features have been repeatedly reported as initial or dominant presentations [4,5]. These scenarios are clinically significant because they occur in environments where copper studies are not routinely requested, and management may focus exclusively on symptom control rather than etiologic clarification [2,5]. In the present case, collateral history revealed a subacute course marked by cognitive-behavioral deterioration and functional decline, supporting an evolving neuropsychiatric process rather than an isolated primary psychiatric disorder.

Interpretation of biochemical findings requires caution. Neuropsychiatric forms of Wilson’s disease may present with only modest abnormalities in routine hepatic tests, and normal or near-normal liver biochemistry does not exclude the diagnosis [1-3]. In this patient, profound hypoceruloplasminemia strongly supported Wilson’s disease, while confirmation of Kayser-Fleischer rings provided a highly specific clinical anchor, particularly given their strong association with neurological involvement [1,2]. Although serum copper was reduced, this finding is compatible with Wilson’s disease and reinforces the importance of integrated diagnostic frameworks that combine clinical features, copper metabolism studies, ophthalmologic findings, and genetic data rather than relying on any single parameter [1,2]. 

Neuroimaging findings should be interpreted as supportive rather than definitive. Educational emphasis on a narrow set of “classic” imaging signs may create false reassurance when these are absent. Large neuroradiological cohorts demonstrate that thalamic and brainstem involvement occurs within the recognized spectrum of neurological Wilson’s disease and may be more frequent in patients with delayed diagnosis [6-9]. Notably, extended diagnostic latency has been associated with higher rates of pontine, midbrain, and cortical lesions [6]. The imaging pattern observed in this patient, bilateral thalamic and brainstem involvement with discrete putaminal changes, fits within these described distributions and further reinforces the importance of early diagnosis to prevent extensive neurological damage. Beyond lesion localization, volumetric and connectivity studies suggest that Wilson’s disease affects distributed cortico-subcortical networks, providing a plausible substrate for the coexistence of motor and psychiatric manifestations [7,8,10].

Therapeutic timing has direct implications for outcome. Copper-lowering therapy remains the cornerstone of management, and penicillamine continues to be widely used within guideline-based strategies [1,2]. Careful monitoring is required during treatment initiation, as neurological worsening has been described in a subset of patients during early chelation [1,2]. In this case, treatment was followed by gradual improvement, supporting the concept that neuropsychiatric dysfunction may be at least partially reversible. Long-term management should integrate mental health follow-up, given the documented impact of psychiatric symptoms on quality of life [11] and should include monitoring strategies designed to avoid both insufficient copper removal and excessive decoppering. Although clinically significant copper deficiency is uncommon, it has been reported and reinforces the need for individualized long-term surveillance [12].

From a practical perspective, this case reinforces several diagnostic cues: acute or atypical psychiatric presentations in young adults, particularly when accompanied by dysarthria, extrapyramidal signs, cognitive slowing, or unexplained functional decline, should prompt consideration of Wilson’s disease and targeted investigation [1-5]. Multidisciplinary collaboration is often necessary to shorten diagnostic latency and optimize outcomes.

## Conclusions

Wilson’s disease can present as a psychiatric emergency and may be misinterpreted as primary mental illness when neurological signs are subtle or overlooked. This case demonstrates that meticulous clinical examination, collateral history, targeted copper metabolism testing, and confirmatory ophthalmologic and genetic assessment can rapidly redirect diagnostic reasoning. Early initiation of penicillamine-based therapy, together with appropriate psychiatric management, can result in meaningful clinical recovery and may reduce the risk of persistent neurological impairment.
